# Extensive Cervico-Thoraco-Abdominal Attacks of Angioedema: CT Diagnosis in Two Unusual Cases

**DOI:** 10.5334/jbr-btr.1275

**Published:** 2017-04-25

**Authors:** Bruno Coulier, Luc Montfort, Lise-Marie Vandezande, Anne-Catherine Bafort, Monica Gogoase

**Affiliations:** 1 Clinique Saint-Luc, Bouge, BE

**Keywords:** Abdominal, CT, Angioedema, Angioneurotic edema, Thoracic, CT, Thoracic duct

## Abstract

Angioedema (AE) classically manifests as an acute transient swelling of extra-visceral spaces, subcutaneous and submucosal tissues. Sometimes it may be a life-threatening condition. The causes are numerous, and the common denominator is an increased vascular permeability allowing diffusion or extravasation of fluid from the vascular bed to the interstitial space. The severity of AE is related to the cause, body location, and extension. We hereby report two very unusual cases characterized by a massive attack of AE from the left cervical area to the pelvis through the length of the mediastinum and axial posterior retroperitoneum. The diagnosis was established by CT. The first case was found related to drug intake, and the second appeared idiopathic.

## Introduction

Angioedema (AE) – first named angioneurotic oedema – is an acute transient phenomenon due to increased vascular permeability allowing diffusion or extravasation of fluid from the vascular bed to the interstitial space [1]. It results from overproduction or failure to inactivate vasoactive stimulants such as histamine, bradykinin, and leukotrienes [2].

The result is classically an acute swelling of extra-visceral spaces, subcutaneous and submucosal tissues lasting two to seven days. In some cases, it may be a life-threatening condition. Severity of AE is related to the cause, body location, and extension. Superficial regions in the face, neck, and upper airways are mostly involved. More rarely, AE may manifest by visceral AE involving the abdominal organs and especially the gastrointestinal tract. Genitourinary involvement may also be rarely encountered [1,3,4].

We hereby report two very unusual cases drastically differing from all classical presentations and in which a massive attack of AE was found extending from the neck to the pelvis through the length of the mediastinum and the axial posterior abdominal retroperitoneum. The full diagnosis was made by CT in the two cases. The first was considered related to drug intake and the second seemed idiopathic.

## Report of Cases

### Case 1

A 45-year-old woman presented to the emergency department with marked clinical swelling of the left supraclavicular fossa. The phenomenon had suddenly developed within a few hours and without any particular effort nor cough or pain. The patient also reported some chest tightness. Physical examination revealed slight left paraombilical tenderness. Basic laboratory tests were irrelevant.

**Figure 1:**
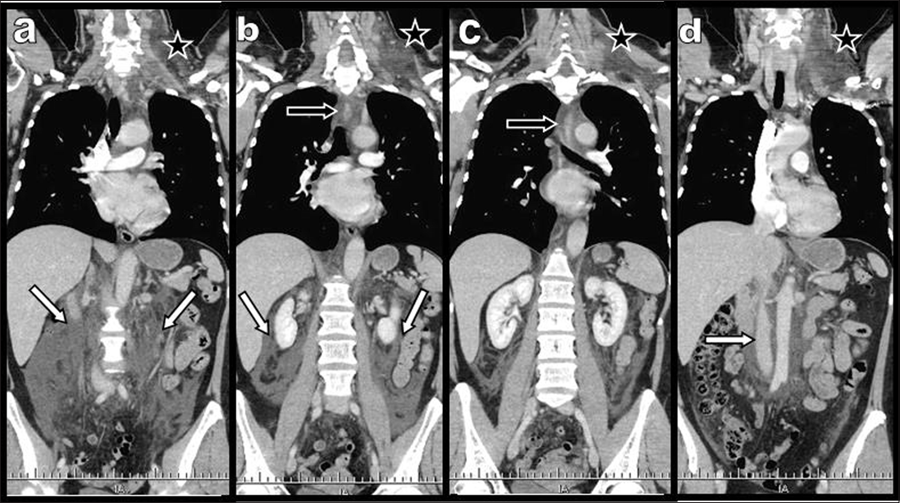
Case 1: Four posterior **(a)** to anterior **(d)** total body global coronal reconstructions obtained during contrast-enhanced thoracoabdominal CT illustrate massive left cervical oedema (black star), diffuse posterior mediastinal oedema (black arrow), and diffuse extensive retroperitoneal oedema (white arrows) extending laterally through the perirenal spaces.

**Figure 2:**
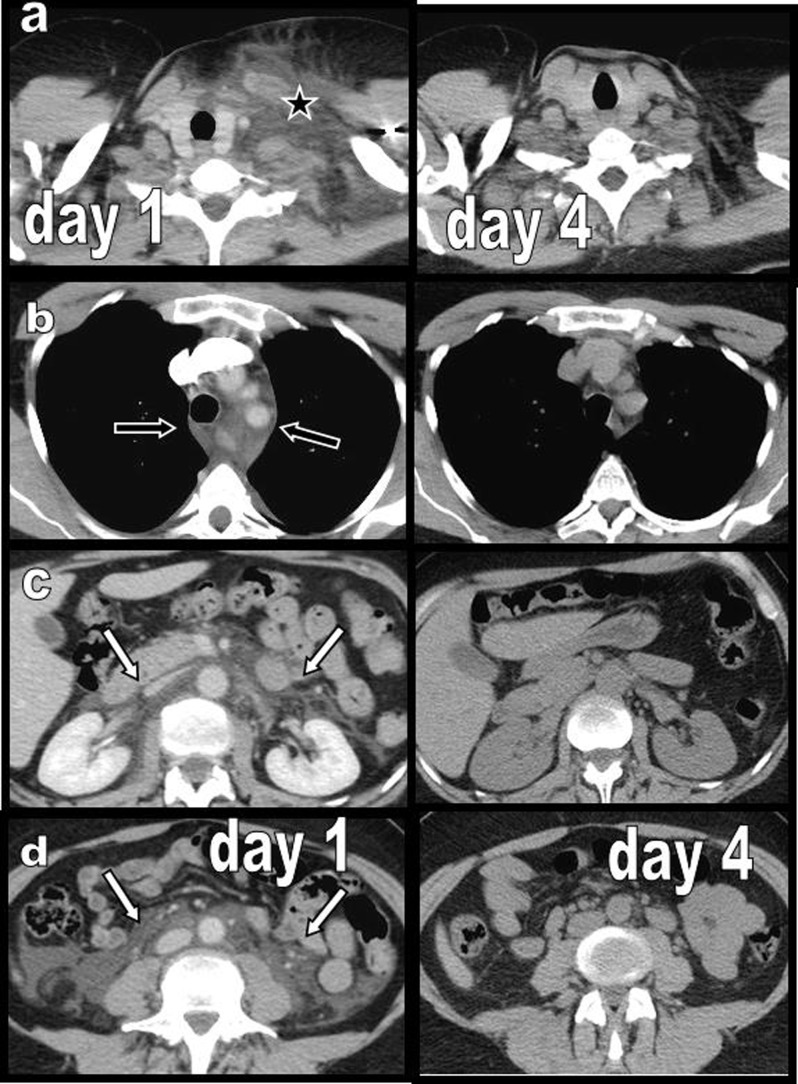
Case 1: Four selected contrast-enhanced CT axial views **(a–d)** illustrating massive left cervical oedema (black star on a), diffuse posterior mediastinal oedema (black arrow on b), and diffuse extensive retroperitoneal oedema (white arrows on c and d) are presented with their corresponding unenhanced views obtained after four days. Massive oedema has completely and drastically disappeared.

On contrast-enhanced thoraco-abdominal CT (Figure 1 and left part of Figure 2), massive and continuous oedematous infiltration extending from the neck to the upper strait of the pelvis was found. Nearly all mediastinal fat tissue was infiltrated, and the abdominal involvement consisted of massive axial posterior retroperitoneal oedema extending laterally through the perirenal spaces. There was no pleural or pericardial effusion nor ascites. Oedema of the gastrointestinal tract was absent. Symptoms spontaneously drastically regressed within the first 48 hours, and an unenhanced follow-up CT performed after four days (Figure 2, right part) confirmed the complete disappearance of oedema. The final proposed diagnosis was an unusual acute massive attack of AE.

Complementary laboratory tests excluded acquired or congenital deficiency of the C1 esterase inhibitor (C1-INH). A possible relation was found with the intake of Biocondil by the patient. Biocondil contains chondroitin and glucosamine, this last component being synthesized from chitin extracted from the crustacean carapace (shrimps, langoustines, crabs, lobsters), a well-known allergenic potential.

### Case 2

A 60-year-old woman was referred to the emergency department with an acute swelling of the left supraclavicular and left laterocervical areas. These complaints were associated with intense abdominal pain. Basic laboratory tests were irrelevant. Anamnesis revealed a previous and spontaneously resolving episode of ascite and left cervical swelling 15 years earlier.

**Figure 3:**
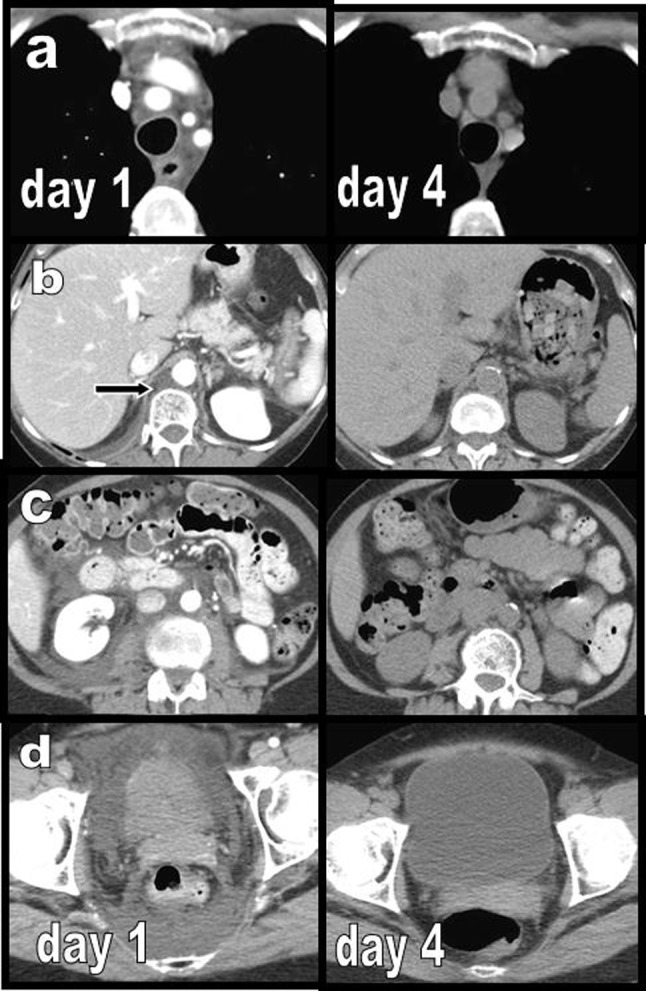
Case 2: Four selected contrast-enhanced CT axial views **(a–d)** illustrating diffuse mediastinal oedema (a) distension of the cisterna chyla (black arrow on b), diffuse extensive retroperitoneal oedema (c), and extensive subperitoneal perirectal and perivesical oedema (d) are compared with their corresponding unenhanced views obtained after four days. Massive oedema has drastically completely disappeared, and the volume of the cisterna chyla has reduced.

**Figure 4:**
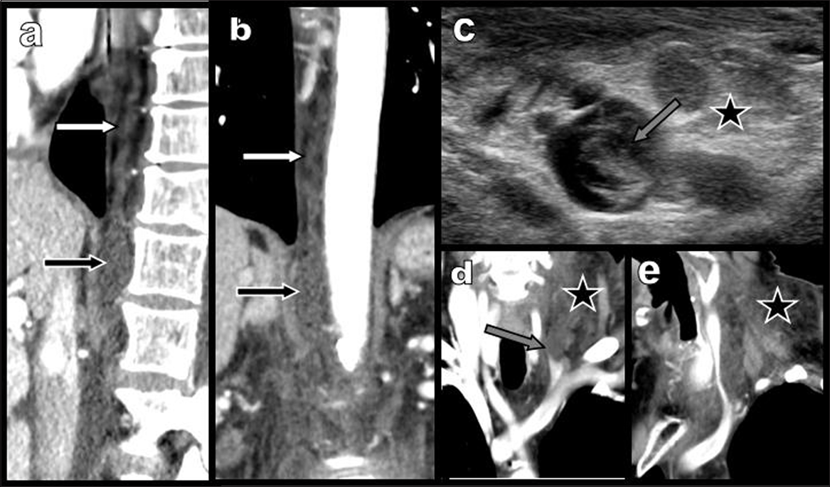
Case 2: Sagital **(a)** and coronal **(b)** reconstructions at the level of the retrocrural spaces of the diaphragm show distension of the cisterna chyla (black arrow) and a large tortuous thoracic canal (white arrow). Transverse ultrasound view **(c)** and the corresponding contrast-enhanced coronal CT reconstructions **(d)** and **(e)** at the level of the swollen supra-clavicular fossa show massive oedema (black star) and deformation and laminating of the left jugular vein by a tortuous hypoechoic serpiginous structure, probably representing the jugular anastomosis of an ectasic congestive thoracic duct (grey arrow).

Cervical ultrasound showed a major dedifferentiation and oedema of the left laterocervical of supraclavicular areas. A heterogeneous tubular structure penetrating the distal internal jugular vein was first considered as a venous thrombosis (Figure 4c). Complementary contrast-enhanced thoraco-abdominal CT revealed massive and continuous oedematous infiltration extending from the neck to the pelvic floor through the mediastinum (Figure 3, left side). The abdominal retroperitoneal extension of oedema was predominant on the right side and around the right kidney and also in the retroperitoneal perivesical and perirectal fat tissue. Additional findings were a very small amount of right pleural fluid and a limited amount of ascite in the right paracolic gutter and in the pelvis. Oedema of the gastrointestinal tract was absent. Distension of the cisterna chyli and of the thoracic canal were visible within the posterior mediastinum (Figure 3b and Figures 4a and 4b).

The tubular structure identified during ultrasound at the junction of the internal jugular and subclavian vein (Figure 4c) was clearly identified on coronal oblique CT reconstructions (Figures 4d and 4e) as the engorged distal and anastomoting portion of the thoracic duct. Symptoms and imaging findings normalized in four days (Figure 3, right side). Complementary laboratory tests excluded acquired or congenital deficiency of the C1 esterase inhibitor (C1-INH).

It was first concluded that the common causal denominator of the two episodes of AE was the intake of diclofenac ®, but another episode of AE occurred spontaneously a few weeks later, nullifying this hypothesis. The AE was thus considered idiopathic, and the patient is now preventively empirically treated with Tranexamic acid (Exacyl ®).

## Discussion

The physiopathological causes of AE are numerous but may pragmatically be classified following their main mechanism resulting from either bradykinin or histamine mediated responses [1,2].

Bradykinin-mediated AE disorders essentially comprise the hereditary AE (HAE) due to a decrease level or a dysfunctional C1 esterase inhibitor (C1-INH), secondary acquired C1 inhibitor deficiency (associated with lymphoproliferative or autoimmune diseases), and specific types induced by various drugs. Angiotensin-converting enzyme (ACE) inhibitors are mostly involved, but also many other drugs. Bradykinin-mediated AE is not associated with urticaria and not mediated by IgE antibodies.

AE mediated by histamine mostly concerns allergic or idiopathic AE – which represents the majority of cases and present with or without urticaria or anaphylaxis. When a trigger is identified, it is most often a drug, an alimentary allergen, latex, or an insect sting [2]. A physical agent may also be implicated, such as cold, pressure, vibration, ultraviolet, etc.

In our first patient, the cause appeared related to drug intake, but no cause was established for the second, which was considered fully idiopathic.

AE have a longer duration (2–5 days) when mediated by bradykinin than histamine (24–48 hours), and classically the first do not respond to antihistamines and corticosteroids [1]. Clinically, AE is defined by episodic transient oedematous swelling affecting any single part of the body or multiple sites simultaneously. Superficial regions in the face, neck, and upper airways are mostly concerned, and secondary upper airways obstruction can be a life-threatening complication [2,3,4]. The differential diagnosis of upper airway obstruction includes neoplasms, abscesses, and infections.

The role of medical imaging is very limited in these types of self-limiting peripheral cases of AE classically involving the face, the neck, and also but more rarely external genital organs or the extremities [2]. Medical imaging nevertheless appears valuable to evaluate the upper airway for obstruction and for the differential diagnosis by exclusion of other neoplastic or infectious processes.

More rarely and sporadically, AE may also involve deep organ systems such as the gastrointestinal tract and the genitourinary tract, even in the absence of peripheral cutaneous involvement and thus without external visible manifestation. The acute or recurrent symptoms are non-specific, comprising severe abdominal pain with distension and tenderness, nausea and vomiting, diarrhoea or sub-occlusion. Acute pancreatitis may be associated due to duct obstruction or oedema of the ampulla of Vater.

Medical imaging is more valuable in these cases [2,3,4,5,6]. Contrast-enhanced CT of the abdomen constitutes the modality of choice and is of primary importance to make the correct diagnosis and especially to rule out all innumerable other causes of acute abdominal pain and to avoid unnecessary surgery [2,4]. According to the same principle, in patients with a previous history of AE who present with complaints of abdominal pain, an abdominal CT is recommended to estimate possible visceral involvement and to exclude other possible surgical pathology.

In typical cases of visceral AE, the affected bowel loops show a typical target or “halo” sign due to a low-density mucosa oedema contrasting with mucosal or subserosal enhancement [4,6]. Free ascitic fluid may be present. Differential diagnosis of typical gastrointestinal AE involves other pathologies causing bowel wall oedema, including ischemia, vasculitis, or hypoproteinemia.

Genitourinary involvement may also be rarely encountered. Renal involvement may simulate renal colic and urinary bladder AE presents with lower abdominal pain and retention. The cardinal signs are oedematous wall thickening with adjacent fluid [2,4].

Our two reported cases drastically differ from all these classical presentations, and to our best knowledge, such massive attacks of “total body AE” have not been clearly reported before, even in a recent review [2].

We have only identified a single case report of isolated and relatively dominantly elective retroperitoneal ACE inhibitor–induced AE [7]. The CT findings of the retroperitoneal oedema appeared very similar to our cases, and the regression of oedema produces promptly three days after responsible drug discontinuation.

Classically retroperitoneal oedema has only sometimes been reported as an associated finding of gastrointestinal visceral AE, but not as an isolated event, as in our patients [7]. An interesting common finding observed in our two cases was the presence of an asymetrical oedema of the left cervico-thoracic junction at the level of the supraclavicular fossa. Clinically, this area appeared swollen and constituted the leading clinical symptom.

This type of swelling was reported by Dendorfer [8] as a recurrent symptom in a 33-year-old patient. Is was considered as an isolated symptom, probably caused by cystic dilatation of unknown origin of the cervical portion of the thoracic duct. Abdominal sonography of the patient was reported being normal. Nevertheless, there was a major discordance with the other symptoms [8] – diffuse abdominal and flank tenderness, fullness, flatulence, increase of waist circumference, and weight gain [8] – all symptoms that could better be compatible with overlooked abdominal extension.

A heterogeneous pseudocystic dilatation of the cervical distal portion of the thoracic duct was observed in our second patient. Nevertheless, this entity was transient and completely disappeared – CT had normalized four days later – thus rejecting the hypothesis of malformation or obstruction of the duct. Moreover, the fact that the full length of the thoracic duct as well as the cisterna chyli were simultaneously dilated could probably be the result of overflow of the lymphatic system caused by the massive mediastinal and retroperitoneal fluid extravasion.

The reports of our patients also drastically differ from the clinical and imaging features resulting from situations of disruption or blockage of the thoracic duct or of its main lymphatic tributaries. The causes of these situations may be tumoral, mechanical but also surgical or traumatic [9,10,11,12]. These situations are nevertheless mostly dominated by manifestations of chylothorax, chylomediastinum, chylous ascites but also sometimes of chylopericardium. Our patients did not have these signs.

To conclude, we reported two rare occurrences of extensive AE. It may be questionable whether the two reported cases are really quite rare and atypical – because of their unusual extensive thoraco-abdominal manifestations – or if they simply correspond to situations that are more frequent but overlooked because of insufficient imaging. Indeed, the dominant clinical symptom of our two patients was a cervico-supraclavicular swelling, thus a mainly peripheral symptom likely to be under-investigated.
